# *In Vitro* Maturation of a Humanized Shark VNAR Domain to Improve Its Biophysical Properties to Facilitate Clinical Development

**DOI:** 10.3389/fimmu.2017.01361

**Published:** 2017-10-23

**Authors:** John Steven, Mischa R. Müller, Miguel F. Carvalho, Obinna C. Ubah, Marina Kovaleva, Gerard Donohoe, Thomas Baddeley, Dawn Cornock, Kenneth Saunders, Andrew J. Porter, Caroline Jane Barelle

**Affiliations:** ^1^Elasmogen Ltd., Aberdeen, United Kingdom; ^2^Molecular Partners AG, Zurich, Switzerland; ^3^Pfizer, Aberdeen, United Kingdom; ^4^Department of Chemistry, University of Aberdeen, Aberdeen, United Kingdom; ^5^UCB BioPharma Sprl, Braine-l’Alleud, Belgium; ^6^Department of Molecular and Cell Biology, Institute of Medical Sciences, University of Aberdeen, Aberdeen, United Kingdom

**Keywords:** VNAR, soloMER, single chain binding domain, shark, humanization, half-life extension, pharmacokinetics

## Abstract

Molecular engineering to increase the percentage identity to common human immunoglobulin sequences of non-human therapeutic antibodies and scaffolds has become standard practice. This strategy is often used to reduce undesirable immunogenic responses, accelerating the clinical development of candidate domains. The first humanized shark variable domain (VNAR) was reported by Kovalenko and colleagues and used the anti-human serum albumin (HSA) domain, clone E06, as a model to construct a number of humanized versions including huE06v1.10. This study extends this work by using huE06v1.10 as a template to isolate domains with improved biophysical properties and reduced antigenicity. Random mutagenesis was conducted on huE06v1.10 followed by refinement of clones through an off-rate ranking-based selection on target antigen. Many of these next-generation binders retained high affinity for target, together with good species cross-reactivity. Lead domains were assessed for any tendency to dimerize, tolerance to N- and C-terminal fusions, affinity, stability, and relative antigenicity in human dendritic cell assays. Functionality of candidate clones was verified *in vivo* through the extension of serum half-life in a typical drug format. From these analyses the domain, BA11, exhibited negligible antigenicity, high stability and high affinity for mouse, rat, and HSA. When these attributes were combined with demonstrable functionality in a rat model of PK, the BA11 clone was established as our clinical candidate.

## Introduction

In recent years, different approaches such as complementarity-determining region (CDR) grafting, framework-homology-based, germline-homology-based re-surfacing, and epitope depletion have been adopted to reduce potential immunogenic responses from non-human therapeutic biologics (1–5). According to Reichert (6), of the 52 antibodies in late stage clinical trials in Europe and the US, the majority, 43, are humanized or are fully human exemplifying the success and clinical validation of these processes. Furthermore, the six monoclonal antibodies (mAbs) approved for use in the European Union or the US in 2016, three were humanized, two were fully human, and one was a chimeric IgG1 with a further 10 human or humanized antibodies in review (6). Of those mAbs approved for therapeutic use, three are the blockbuster drugs for oncological indications, Rituximab (Rituxan), Trastuzumab (Herceptin), and Bevacizumab (Avastin), each of which generated in excess of $6 billion in revenues in 2015 (7–9).

The development of next-generation therapeutic biologics has been gaining pace with over 50 products based on both immunoglobulin and non-immunoglobulin scaffolds at varying stages of development (6, 10). Much of this development reflects the drive to overcome the limitations of classical antibodies such as their complex structure, and large size resulting in high cost of manufacture from an economic perspective and limited tissue penetration from a biological perspective (11–14). The focus of this study was the improvement of the biophysical properties of a humanized VNAR, which is the variable binding antigen specific domain derived from the new immunoglobulin antigen receptor or IgNAR (15). As IgNAR forms an integral part of the adaptive immune system of sharks, high-affinity, highly selective IgNARs can be raised through immunization and target-specific VNAR domains selected through classic phage display technology (16–18). Although demonstrating the target-specific attributes of antibodies, VNARs do not originate from an immunoglobulin lineage but are postulated to have evolved from cell surface members of the Ig superfamily such as ICAMs and VCAMs (19–21). Interestingly, their sequence identity is closer to human light chain frameworks than heavy chains. As with other non-human sources of antibodies, this reduced relatedness to human mAbs is the driving force behind developing a humanization strategy for the development of these domains for clinical use. Elasmogen Ltd. has a proven, patented methodology for the humanization of VNAR domains, the endpoint of which are therapeutic clinical candidates known as soloMERs™ (22).

The subject of this work, hE06v1.10, is a humanized version of an anti-HSA clone called E06 that was originally isolated from an immunized dogfish (18, 23). E06 can be fused with many different therapeutic partners including but not limited to, VNAR domains, scFvs, peptides, and proteins to increase their systemic half-life and subsequent therapeutic window (23). With broad utility across multiple indications, the biophysical properties of the final humanized version of E06 were critical to negate any downstream production issues with aggregation or potential immunogenicity. The first steps toward humanization of VNAR domains were conducted by Kovalenko et al. (24) using the anti-hen egg (HEL) domain, 5A7 (16), as a model template. Given the evolutionary distance between VNARs and true IgGs, the foundation of the approach was based primarily on structural similarity to human framework scaffolds. Using this strategy, DPK9 was selected. The underlying methodology used to successfully humanize 5A7 was then transposed onto the anti-human serum albumin (HSA) binding VNAR domain, E06 resulting in the construction of v1.10. A second version of humanized E06, v2.4, was designed based on human Kappa germline framework DPK24.

This work has studied v1.10 and v2.4 in more depth and has revealed a propensity for the v1.10 domains to dimerize and in the case of v2.4 to bind to HSA with lower affinity, questioning their applicability for clinical development. To overcome the unfavorable characteristics of v1.10 and v2.4, these were used as templates for mutagenesis to select better performing humanized versions of these proteins.

## Materials and Methods

### Random Mutagenesis of E06v1.10 and E06v2.4

E06v1.10 and E06v2.4 sequences were cloned into the phage display vector pWRIL-9 and tested as periprep extracts for binding to cognate target and relevant controls. This vector is comparable to pWRIL-1 (25) with the c-Myc tag replaced by an HA tag, and the leader sequence is derived from pelB. Sequences were mutagenized by error-prone PCR to deliver up to 9 substitutions/VNAR sequence using a GeneMorph II random mutagenesis kit (Agilent Technologies, Santa Clara, CA, USA). Libraries were cloned into pWRIL-9 and transformed into TG1 electrocompetent cells (Lucigen, Middleton, WI, USA). Library diversity was analyzed by sequencing over 100 clones from each repertoire.

### Selection from Libraries by Phage Display

Libraries were rescued and selected twice using Nunc Maxisorp immunotubes as previously described (26). For the first round of panning, tubes were washed five times with PBS containing 0.1% Tween 20 (PBS-T) and five times with PBS; for pan 2, the number of washes with and without Tween 20 was doubled. Following each round of panning, two 96-well plates of individual colonies were picked with a QPix2 XT (Genetix, San Jose, CA, USA) and grown for periplasmic protein extraction. Binding to HSA (and HEL control) was evaluated by ELISA using 50% of the crude periplasmic protein extract (27). VNARs were detected via their HA tag using a high-affinity mAb HRP conjugate (clone 3F10; Roche, Basel, Switzerland). All samples were processed with a Perkin Elmer MiniTrak robotic liquid handling system (Waltham, MA, USA).

### Reformatting to Eukaryotic Expression Vector and Analysis of Output by ELISA

Unique clones showing OD_450_ by periprep ELISA at least 25% higher than the readings obtained from parental huE06v1.10 or v2.4 were selected for batch conversion into the proprietary mammalian expression vector pSMED2. Equal amounts of pWRIL-9 plasmid DNA from clones were pooled, and VNAR sequences were amplified by PCR with primers inserting *Bss*HII and *Eco*RI at the 5′ and 3′ ends, respectively. After cloning and transformation of *Escherichia coli* TG1 electrocompetent cells, a fourfold over-representation of the starting number of clones was grown in 96-deepwell culture plates (Greiner Bio One, Frickenhausen, Germany) and plasmid DNA purified using a QIAprep 96 Turbo BioRobot Kit in a BioRobot 8000 (Qiagen, Hilden, Germany). In a 96-well plate format, each clone was expressed transiently in 200 µL of HEK293 cells previously adjusted to a density of 10^6^/mL. Each 200 µL culture was transfected with 200 ng of plasmid DNA using lipofectamine (Invitrogen, Carlsbad, CA, USA) and grown at 37°C and 8% CO_2_ while shaking at 250 rpm to maintain cells in suspension. After 24 h, cultures were supplemented with tryptone to a final concentration of 0.5% and expression continued for 6 days. Post-expression media samples from HEK 293 transfections were tested for binding to HSA and HEL by ELISA. Detection was achieved via an anti-6-His HRP conjugate (ab1187; Abcam, Cambridge, UK).

### Off-Rate Selection Screening

Samples of the “best-performing” media from the small scale HEK293 transfections were subject to kinetic analysis using a T200 BIAcore instrument for off-rate ranking (28) (GE Healthcare Life Sciences, Little Chalfont, UK). For off-rate screening samples were diluted 1:5, 0.2 μm-filtered, then filtrates run over a research-grade carboxy-methyl-dextran chip (CM5) onto which HSA was immobilized using standard amine coupling chemistry. The association phase for all samples was 2 min, and the dissociation was monitored for 3 min at a flow rate of 100 µL/min, followed by two 10 µL injections of glycine pH 1.5 at a flow rate of 100 μL/min. All binding experiments were performed at 25°C in HBS/EP buffer. Analysis of the resultant sensorgrams made use of the 1:1 global Langmuir binding model. Those samples showing the slowest dissociation rates were then selected for larger-scale protein production and DNA sequences of VNARs determined.

### ELISA Assay and EC50 Determination

Rat, mouse, and HSAs used in ELISA-binding assays were from Sigma-Aldrich. For direct ELISA formats Nunc Maxisorp 96-well plates were coated at 1 µg/mL antigen in PBS and then blocked with 4% non-fat milk in PBS. Purified 6-his-tagged control and humanized VNAR proteins in PBS were diluted 1/3 into wells and double-diluted further across the plate. After incubation for 1 h, plates were washed three times with 0.05% Tween 20 in PBS. The detection of antigen bound VNARs was achieved by incubation with an anti-6-His HRP mAb for 1 h or where appropriate with an anti HA tag mAb HRP conjugate (clone 3F10; Roche) and developed by adding TMB substrate. When fully developed, the reactions were halted by the addition of 1 M H_2_SO_4_ absorbance measured at 450 nM. Data were processed using SigmaPlot 9.

### Affinity Measurements

Affinities of selected clones were determined on a T200 BIAcore surface plasmon resonance instrument essentially as described previously (24). As well as measuring affinity for HSA, the mutated anti-HSA VNARs were also assessed for binding to mouse serum albumin (MSA) and rat serum albumin (RSA). A CM5 chip was prepared in which the first flow cell was used as a reference to correct for bulk refractive index, matrix effects, and non-specific binding. Approximately 300 RU of HSA was immobilized onto flow cell 2, 350 RU of MSA was immobilized onto flow cell 3, and 600 RU of RSA onto flow cell 4. Prior to immobilization, the serum albumins were made up in 10 mM sodium acetate buffer (pH 4.5) and post-coupling the remaining activated groups was blocked with 1.0 M ethanolamine-HCl pH 8.5. For affinity measurements, purified anti-HSA VNAR monomers, dimers, and trimers were diluted to 1.56–100 nM in HBS/EP buffer and injected over the chip as above. Analysis of the resultant sensorgrams was performed using the 1:1 global Langmuir binding model fit analysis (BIAcore Evaluation Software).

### Protein Expression and Purification

Expression of VNAR proteins for periprep screening and phage ELISA was carried out as described (29). For small-scale transient expression and off-rate screening, mutated VNAR genes were cloned into pSMED2 vector containing a CMV promoter and a C-terminal 6-His tag. Following off-rate screening plasmid preparations of selected clones were scaled up and used for 1 L PEI-mediated transfection and transient expression in HEK293 host cells (30, 31) using serum free FreeStyle™ 293 media (Invitrogen). Purification of expressed protein was achieved by immobilized metal chelate chromatography using Ni^2+^ charged resin followed by cation exchange chromatography with buffer exchange as appropriate between steps. Final protein samples were buffer exchanged to PBS and stored frozen at −20°C. If required, proteins were then subjected to a final polishing step by preparative size-exclusion chromatography using a Superdex 200 26/60 size-exclusion column equilibrated with PBS. Eluted peaks from this chromatography were pooled, then concentrated using Amicon Ultra filtration units. Protein concentrations were determined by UV spectroscopy. Expression levels of VNAR proteins were generally in the region of 0.5–3 mg/L, and electrophoretic analysis of protein samples was performed on 12% NuPAGE BisTris gels using a MOPS buffer system (Invitrogen). Analytical size-exclusion chromatography was performed using an Agilent 1200 series HPLC system and ZORBAX GF 250 9.4 mm × 250 mm 4 μm column or TSK gel G3000PW 7.5 mm × 30 mm column equilibrated with phosphate-buffered saline at pH 7.4 with instrument set up and run parameters adjusted as required for each run.

### Cloning of VNAR Dimeric and Trimeric Constructs

VNAR E06 and lead humanized clones BA11, BB11, and BB10 were selected as the backbone of several dimeric and trimeric fusion protein constructs. Dimers were assembled using a standard PCR overlapping extension techniques joining the albumin binding domain, via a (GGGGS)_4_GAHS flexible linker to the carboxyl or amino terminal end of a control and naive VNAR domain known as 2V. The trimeric constructs were made by flanking the albumin binding domain with the same naive 2V domain at both terminal ends (23). Constructs were cloned into pSMED2, plasmid preparation scaled up and the resultant DNA used to transiently express protein in HEK 293 cells (30, 31).

### Dendritic Cell–T-Cell (DC–T) Assay

A DC–T proliferation assay was used to identify the presence or absence of possible T-cell epitopes within the wild-type E06 and humanized variants 2G, BA11, BB11 and BB10 (performed at ProImmune Ltd., UK using ProImmune’s REVEAL^®^ Immunogenicity System DC–T cell assay). For this study, test proteins and controls were incubated with CD8^+^ T-cell-depleted peripheral blood mononuclear cells (PBMCs) prepared for 7 days, from a panel of 20 different healthy human donors. Each PBMC sample was HLA-typed and donors were selected (by DRB1 allotyping) to approximately represent MHC class II allele frequency distributions across the global population.

In brief, adherent donor PBMCs were cultured with appropriate growth factors to generate monocyte-derived DCs. DCs were then loaded with either test or control antigen protein or left untreated. Test proteins were loaded at a final concentration of ~0.34 μM (5 µg/mL). Mature antigen-loaded DCs were then co-cultured (at a set ratio) with autologous CFSE-labeled T cells in multi-well plates. Each test condition was set up in octuplet and incubated for 7 days. Positive control antigens used for this assay were Tuberculin purified protein derivative (PPD from *Mycobacterium*) at a final assay concentration of 0.2–0.4 µM (~5 μg/mL) (70–100% of donors are expected to react to this protein as a result of previous vaccination, through a memory immune response). A second control antigen used, keyhole limpet hemocyanin (KLH) is a known potent naive protein immunogen. This was used at ~0.64 μM (0.25 mg/mL) in the assay (up to 70% of donor samples might be expected to react to this protein, driven by a naive immune response). At the end of the incubation period, cell samples were stained with anti-CD4 antibody, then washed and fixed for flow cytometric analysis. Proliferation was determined by measuring a decrease in CFSE intensity.

### Pharmacokinetic (PK) Studies

Three groups of four male Sprague Dawley rats received a single intravenous (bolus) administration of 2V-E06-2V, 2V-BA11-2V, or 2V-BB11-2V dosing at 1 mg/kg. Blood samples (0.3 mL) were collected into tubes containing Lithium Heparin as an anticoagulant before dosing and at the following times: 30 min and 4, 12, 24, 48, 84, 96 120, and 144 h. These were placed on ice and plasma collected from each after centrifugation at 3,000 × *g*, at 4°C for 10 min. Prior to analysis plasma samples were stored at −80°C.

### LC–MS/MS Methodology for the Bioanalysis of PK Samples

A quantitative LC–MS/MS method had been previously developed to specifically measure concentrations of 2V-E06 in rat plasma (23). In summary, the method utilizes the C-terminal 6x His tag present on these proteins molecules to provide sample enrichment using a magnetic Ni-NTA bead capture step. Following analyte enrichment and tryptic digestion of the sample, targeted LC–MS/MS is used to quantify specific signature peptides in all parts of 2V and the humanized E06 domains. Signature peptides within each partner were identified by *in silico* tryptic digestion. For E06 and humanized variants BA11 and BB11, the signature peptide EQISISGR was selected and AQSLAISTR for 2V. Control signature peptides and a labeled internal standard peptide EQI-[U13C3, 15N-Ser]-ISGRAQS-[U13C6, 15N-Leu]-AISTRHHHHHH were synthesized (by Cambridge Research Biochemicals, Billingham, UK). Assessment of peptides allowed the PK determination of the VNARs (serum half-life) as well as monitoring of (GGGGS)_4_GAHS linker stability.

### Plasma Pull Down and Trypsin Digestion

VNAR 2V-E06-2V protein was diluted into heparinized rat plasma at 30, 20, 10, 5, 3, 1, 0.3, 0.2, and 0.01 µg/mL. A total of 10 µL of each dilution was added to 40 µL of 0.1 M phosphate buffer pH 8 followed by 50 µL of 6 M guanidine hydrochloride containing 1 µg/mL of the heavy isotope labeled internal standard peptide EQI-[U13C3,15N-Ser]-ISGRAQS-[U13C6,15N-Leu]-AISTRHHHHHH. After mixing, these samples were each reduced and alkylated as previously described (23). For the PK sampling time points, 10 µL of plasma was added to 40 µL of 0.1 M phosphate buffer pH 8. The samples were then diluted by the addition of 100 µL 100 mM phosphate, pH 8, 0.1% CHAPS in preparation for sample enrichment. Sample enrichment and magnetic bead processing was performed in 1.5 mL capped tubes and magnetic capture blocks. Twenty-five microliters Ni^2+^-NTA magnetic beads plus 75 µL 100 mM phosphate, pH 8, 0.1% CHAPS were added to each sample. After mixing, the magnetic beads were trapped by magnetic block and the fluid containing the unbound proteins was removed. The beads were washed three times with 500 µL 100 mM phosphate, pH 8, 0.1% CHAPS with magnetic immobilization of beads between each wash before a final 500 µL wash with 100 mM phosphate, pH 8, 0.1% CHAPS containing 20 mM imidazole. Bound proteins were eluted in 100 µL of 0.5 M Imidazole, 10 mM CaCl_2_, 50 mM Tris HCl, pH 8. Eluted protein samples were then trypsin digested by addition of 20 µL of proteomics grade trypsin (made up at 20 µg/mL in 10 mM CaCl_2_, 50 mM Tris HCl, pH 8) followed by incubation at 37°C for 18 h prior to LC–MS/MS analysis.

### LC–MS/MS Analysis

The samples were loaded into the auto-sampler module of a Waters Acquity UPLC (chilled at 6°C) and 5 µL of the extracted samples injected onto the LC–MS/MS system. The native and stable-labeled signature peptides were separated on Acquity UPLC BEH C18 1.7 µm 2.1 mm × 50 mm column equilibrated with solvent A water/acetonitrile/formic acid (95/5/0.1) and eluted with a stepped gradient as follows. At a flow rate of 0.5 mL/min, the column was washed with buffer A for 4 min, then from 4 to 8 min buffer was changed by gradient mixing to 20% solvent B + 100% acetonitrile + 0.1% formic acid. From 8 to 8.5 min solvent B increased to 55% then back to 45% solvent B at 9 min and back to 100% solvent A at 10 min in preparation for the next sample injection. Chromatography runs were carried out at ambient temperature, and under these conditions, the retention time for the analytical and reference peptides was 6.11 min for EQISISGR and 6.27 minutes for AQSLAISTR (±0.5 min). The peptide analytes EQISISGR and AQSLAISTR and control internal standard labeled peptides were detected by atmospheric pressure electrospray ionization MS/MS using a Xevo TQS MS/MS detector. The analytical column eluate was delivered into the source operated at an IonSpray voltage of 3,300 eV with settings as follows: cone (V) 25, source offset (V) 45, source temperature 150°C, desolvation temperature (600°C), cone gas flow (L/h) 0, desolvation gas flow (mL/min) 0, collision gas flow (mL/min) 0.15, and nebulizer gas flow (Bar) 7.00.

For the analytes and control labeled internal standard peptides, MRM transitions were 445.2–519.3 for EQISISGR, 473.8–747.4 for AQSLAISTR, 447.2–523.3 for EQISISGR-IS, and 477.3–754.4 for AQSLAISTR-IS. In all experimental runs, a system suitability test was performed by the injection of a 50 µg/mL AQSLAISTR and EQISISGR mixed standard in buffer. The ion chromatograms were quantified by reference to standard curves spiked into fresh control plasma and analyzed over the range 0.01–30 µg/mL and a calibration curve constructed by plotting the peak area ratio of the calibration standards *vs*. the concentration of peptide fragments in a control matrix and determine the linear regression parameters of the curve, using a 1/*x*^2^ weighting factor. The concentration of peptide fragments in the quality control and test samples were determined by interpolation of the peak area ratios from the calibration curve.

### Stability Assay

The stability of VNAR E06 and humanized E06 BA11 was assessed after exposure to extremes of temperature and pH. Samples of E06 and humanized E06 BA11 proteins were prepared at 10 µg/mL working concentration and placed on a 100°C preheated block. Samples were withdrawn from experimental conditions at 0, 5, 10, 20, 30, 40, 50, and 60 min time points and transferred into wells containing appropriate volume of PBS pH 7.4 to obtain a final concentration of 0.5 µg/mL. Boiled samples recovered in PBS were kept on ice for 1 h before assessing VNAR E06 and humanized VNAR BA11 in an has-binding ELISA. In a similar assay the pH stability of VNAR domains was assessed. Samples were prepared and incubated at a working concentration of 10 µg/mL in a final volume of 50 µL at the designated pH value. For acidic conditions, pHs 1.5, 3, and 5.5 protein samples in PBS pH 7.4 were adjusted using 1 M HCl or 0.1 M citric acid and for basic pHs 8.5, 10 and 11 samples in PBS were titrated to required pH value using borax buffer or 1 M NaOH. Samples were incubated at room temperature, and aliquots withdrawn at stipulated time points and neutralized in PBS pH 7.4 to a final concentration of 0.5 µg/mL. Samples were incubated at designated pH values for up to 28 days, and the HSA-binding activity of the treated samples was determined using ELISA.

## Results

### Affinity Maturation of Humanized VNARs by Random Mutagenesis, Library Screening and Selection

**Figure 1:**
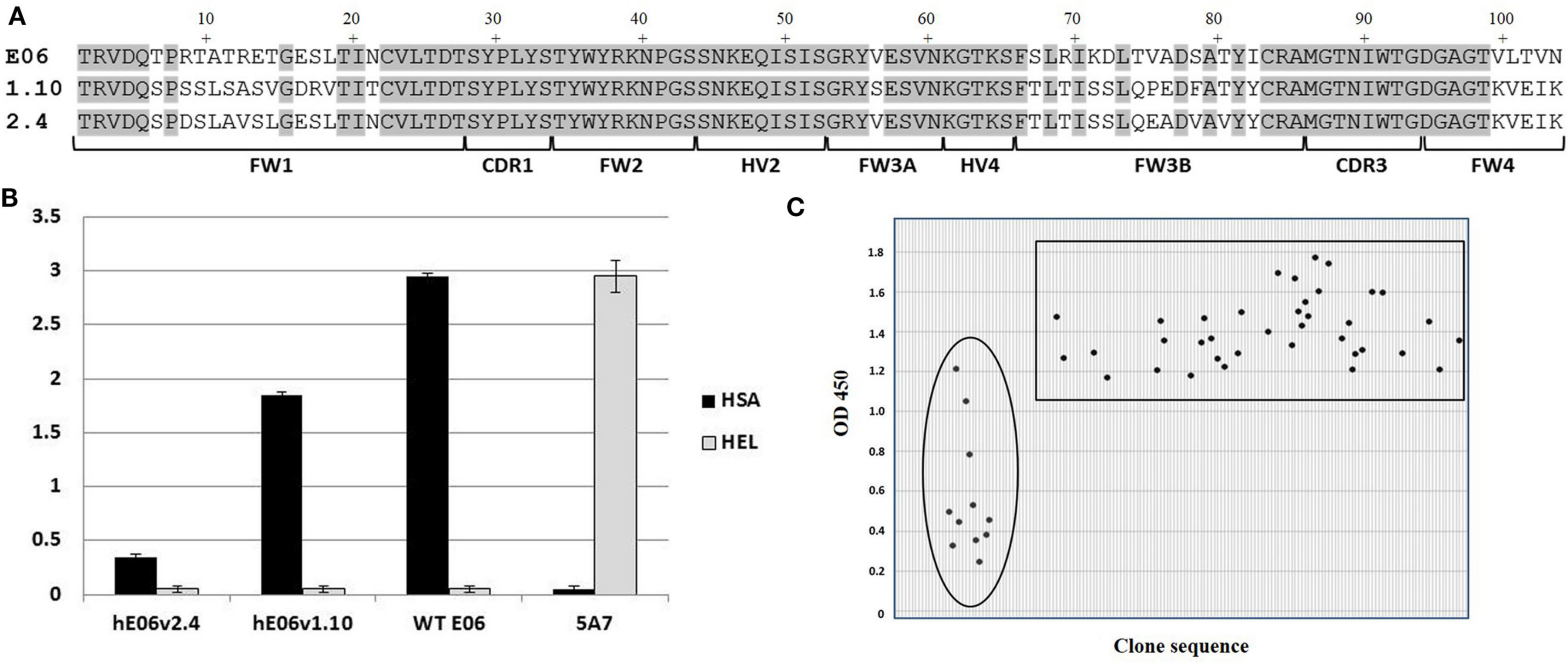
Mutated library design, QC and outputs. **(A)** Amino acid sequence alignment of humanized E06 v1.10 and E06 v2.4 with wild-type E06. FW is framework region; CDR is complementarity-determining region; HV is hypervariable region. **(B)** Phage-binding ELISA to human serum albumin (HSA) and hen egg white lysozyme (HEL)-coated plates. Phage displaying VNAR domains E06, humanized E06 v1.10 and humanized E06 v2.4, and a negative control clone 5A7. Control 5A7 is a hen HEL-binding VNAR. **(C)** Selection of mutated clones based on periprep ELISA binding to HSA. A total of 37 humanized E06v1.10 (boxed) and 12 humanized E06 v2.4 clones (circled) clones were identified for further studies. Criterion for selection was an OD450 (after 5 min development time) of at least 100% of the signal generated by parental humanized E06 v1.10 and E06 v2.4 controls assayed under the same conditions.

**Figure 2:**
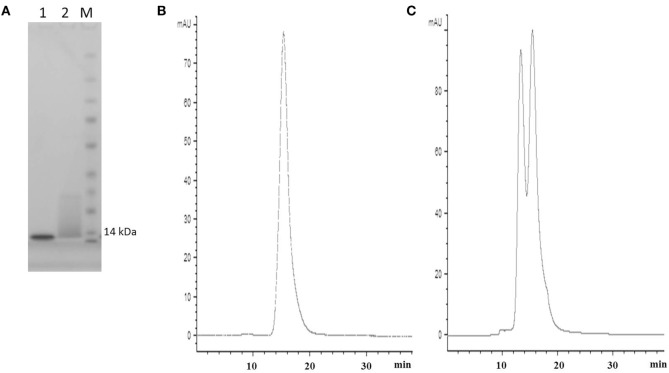
SDS PAGE and SEC profiles of native E06 and humanized E06 v1.10. **(A)** SDS PAGE and Coomassie blue staining of 2 µg of wild-type E06 and E06 humanized v1.10 proteins. Lane 1, E06; Lane 2, E06 v1.10; Lane M, molecular weight markers. **(B)** Analytical SEC chromatogram of wild-type E06 protein. **(C)** Analytical SEC chromatogram of humanized E06 v1.10 protein.

Analysis of E06 clones humanized (hE06) by targeted insertion of residues and sequences from human V Kappa germ lines led to two “parental” molecules (v1.10 and v2.4 Figure 1A) with specific but lower binding affinities and undesirable biophysical properties (Figures 1 and 2 respectively). In order to make improvements to these two humanized variants, two randomly mutated libraries were constructed by error-prone PCR using DNA from these clones as the initial template (v1.10 and v2.4 Figure 1A, based on DPK9 and DPK24 human Kappa germline sequences, respectively). To ensure full coverage of the hE06 sequences and to reap any potential benefits of multiple substitutions in the protein sequences, conditions were optimized to deliver a maximum of nine nucleotide mutations per VNAR. Phagemid libraries of ~5 × 10^7^ clones for both hE06v1.10 and v2.4 were constructed and diversity determined by randomly selecting and sequencing of over 100 individual clones from each library. Results indicated a good coverage of mutations along the full sequence length, with an overall mutation rate of ~90% (90/103 and 94/103 clones had a changed amino acid residue, for hE06v1.10 and v2.4, libraries respectively).

In order to recover molecules with improved binding properties two rounds of phage display selection were performed using HSA as a target. Approximately 200 individual clones were randomly picked from each library and round of panning (total of 800 clones) and tested for binding by ELISA using VNAR protein derived from crude periplasmic extracts. Additional control screening was performed using non target antigens (HEL and blocking agent) to confirm that there was no overall gross VNAR misfolding leading to polyreactivity or non-specific stickiness. An initial examination of the sequences of the enriched clones did not offer up a clear pattern or any obvious positional bias but placed the mutations randomly throughout the parental templates. In addition, parental hE06v1.10 was recovered a total of 53 times from pan 1 and 80 times from pan 2, while hE06v2.4 was observed only once in pan 2 and did not occur in the pan 1 sequences at all. The remaining “new” clones were subjected to further analysis including their expressability and any propensity to form dimers (results not shown). Figure 1C shows the isolation of a final panel of mutated clones based on periprep ELISA binding from which 37 humanized E06v1.10 (boxed) and 12 humanized E06v2.4 clones (circled) were selected for further characterization. These 49 clones were transferred to a eukaryotic expression vector and small scale transient expressions performed in HEK 293 cells.

### Protein Expression, Assessment, and Characterization

**Figure 3:**
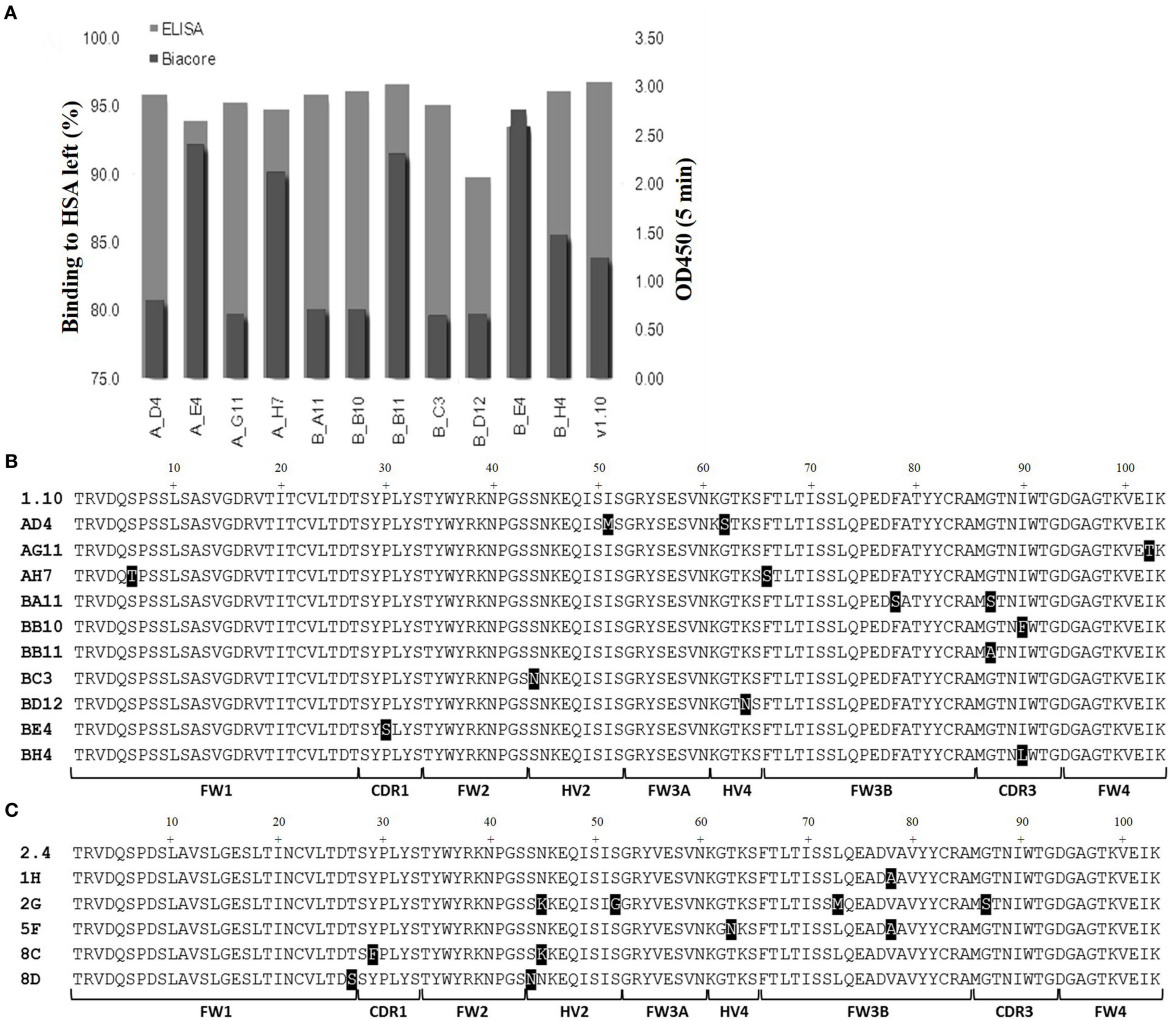
ELISA and off-rate screening analysis of related and humanized mutant. The intensity of binding of media samples from small scale transient expressions in HEK 293 cells were screened and compared by kinetic off-rate analysis, using a T200 BIAcore instrument, and HSA-binding ELISA. **(A)** Selected lead clones derived from v1.10 library (data for v2.4 selection not shown). Off-rate as a percentage of the parental E06 off-rate plotted together with ELISA binding. **(B)** The amino acid sequence of selected clones from the v1.10 mutagenesis library. **(C)** The amino acid sequence of selected clones from v2.4 mutagenesis library. Mutated residues differing from parental sequence are highlighted. See Figure 1 legend for list of abbreviations.

Media samples from HEK 293 expressions were screened for specificity using an HSA-binding ELISA and off-rate ranking was performed on the same samples using a T200 BIAcore surface plasmon resonance instrument. Clones that showed a slow-off rate nearing that of parental E06 and were positive by ELISA, giving signals similar to the parental hE06v1.10 or v2.4 clones, and were selected for further study (Figure 3A). The sequences of these selected clones were determined and those from v1.10 and v2.4 derived libraries are shown in Figures 3B,C, respectively. The positions of the mutations identified, even in this focused sub-panel of lead clones, were typically one or two amino acid mutations randomly located along the length of the VNAR proteins (Figures 3B,C).

**Figure 4:**
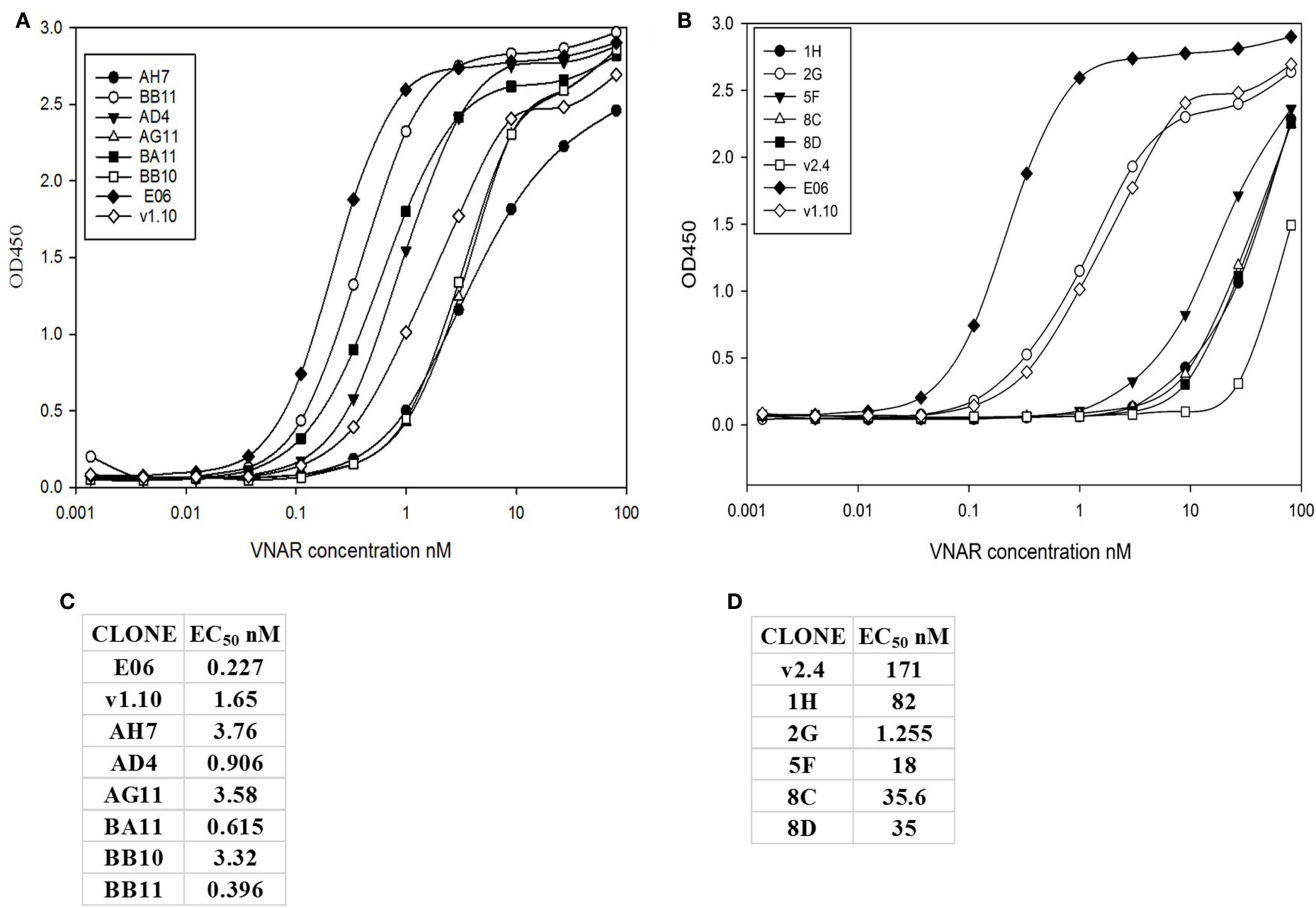
ELISA and EC_50_ determination of lead v1.10 and v2.4 humanized E06 clones. Purified E06 His-tagged VNAR protein and selected lead clones were added to wells of an human serum albumin-coated plate at a concentration of 1 µg/mL and serially diluted threefold across the plate. Four parameter logistic curve, adjustments and EC_50_ calculations were performed with SigmaPlot 9.0. **(A)** Binding curves of selected lead clones derived from mutagenesis of the v1.10 backbone. **(B)** Binding curves of selected lead clones derived from mutagenesis of the v2.4 backbone. **(C)** EC_50_ values of lead v1.10 derived clones. **(D)** EC_50_ values of lead v2.4 derived clones. With the exception of E06 v2.4-2G, clones obtained by mutagenesis of E06 v1.10 performed better with several selected clones showing single digit nanomolar or lower EC_50_ values.

Transient expression of these 15 lead clones was scaled up in HEK 293 cells to produce proteins for further study. After scale up and growth in serum free media and post-purification, expression levels of between 3 and 10 mg/L were obtained for the humanized monomeric, dimeric, and trimeric constructs. Purified protein samples were quantified by UV spectroscopy and concentrations determined. For the best expressing clones, together with suitable controls (E06 wild-type, v1.10, AH7, AD4, AG11, BA11, BB11, BB10, and v2.4 derived clones 1H, 2G, 5F, 8C, and 8D), ranking of the purified proteins was determined by ELISA (Figures 4A,B) and EC_50_ values calculated using SigmaPlot 9.0 (Figures 4C,D). Based on expression levels and EC_50_ values, a final panel four of clones was selected. From the mutated v1.10 library BB10, BA11, and BB11 and from the v2.4 derived library, clone 2G was characterized in more detail.

### SDS PAGE and Analytical SEC of Final Four Purified Leads

**Figure 5:**
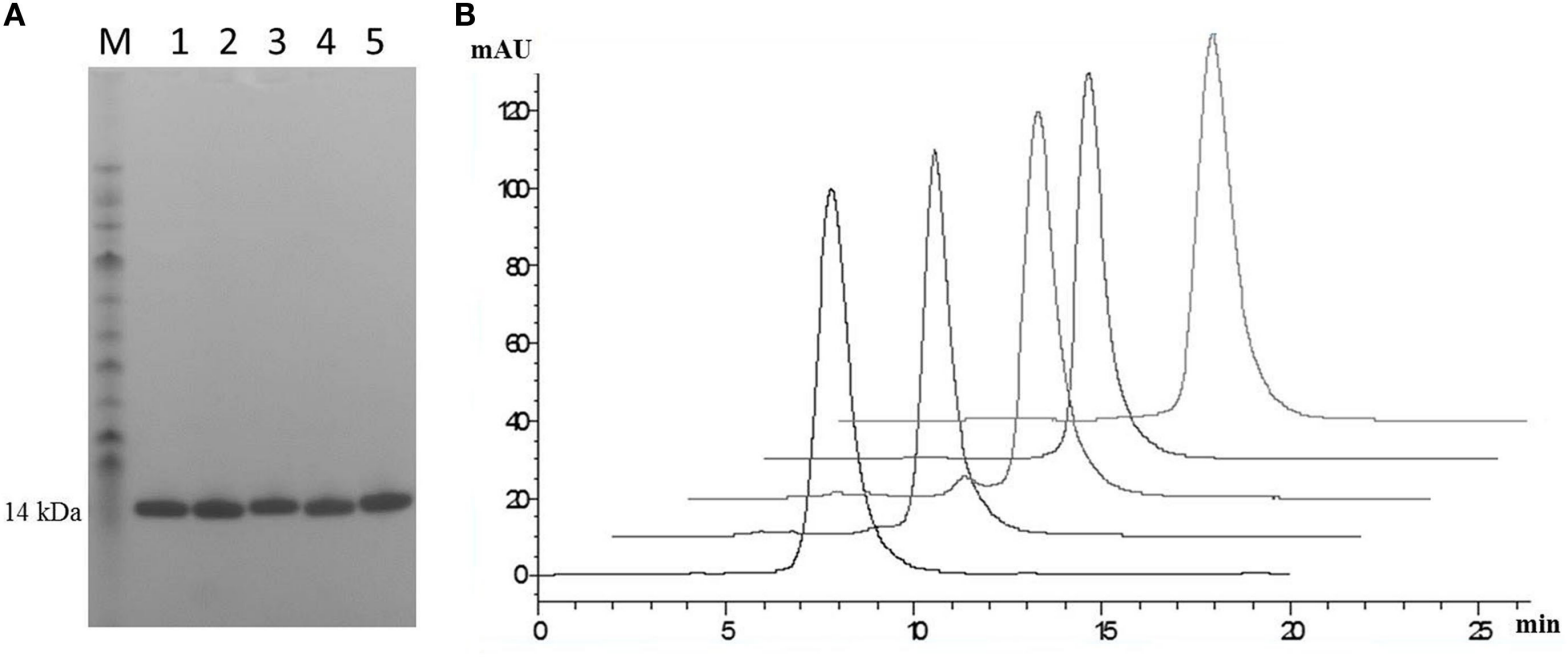
SDS PAGE and analytical SEC analysis of purified lead humanized E06 proteins. **(A)** Samples of purified humanized VNAR protein analyzed by SDS PAGE. Lane M, molecular weight markers; Lane 1, BB10; Lane 2, BA11; Lane 3, BB11; Lane 4, E06 and Lane 5, 2G. **(B)** Analytical SEC profiles of purified lead monomer proteins overlaid and offset to aid comparisons. Profiles are from left to right: BB10, BA11, BB11, E06, and 2G. Data from both analytical techniques confirms that proteins tested appear to be monomeric in nature.

Post-purification proteins were analyzed by SDS PAGE and analytical SEC. On SDS PAGE, the selected clones ran as a single band (Figure 5A), with an expected molecular mass of 14 kDa. Analytical size-exclusion chromatography of the purified monomeric BB10, BA11, BB11, 2G, and E06 control appeared mainly as single peaks showing minimal signs of dimerization (Figure 5B) unlike the parental clone v1.10 (Figure 2C). The purified BB11 monomer showed a small peak eluting from the column ahead the of the main monomeric protein peak. It is unclear if this is dimerized BB11 protein or low level higher molecular weight contaminating protein. The remaining humanized VNAR monomers eluted as single peaks.

### Antigenicity Assessment of E06 and Four Lead Humanized VNAR Clones

**Figure 6:**
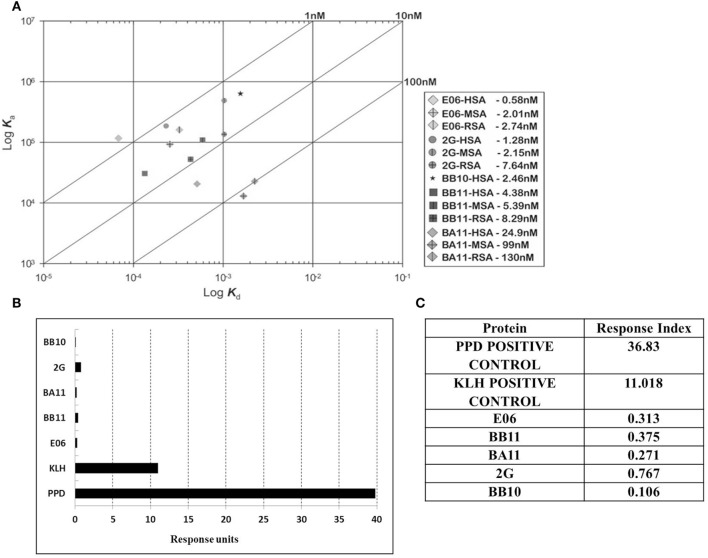
Affinity measurement and antigenicity assessment of lead humanized VNAR albumin binding domains. **(A)** Kinetic distribution plot and BIAcore affinity values for purified monomeric proteins E06, BB10, BA11, BB11 and 2G specific for human serum albumin, rat serum albumin, and mouse serum albumin. Values are the average of at least two experimental runs. **(B)** Proliferative T-cell response used to assess immunogenicity of monomeric E06, BB10, BA11, BB11, and 2G. **(C)** Table of calculated response index values. Positive control antigens used for the assay were (i) Tuberculin purified protein derivative (PPD from *Mycobacterium*) at a final assay concentration of 5 µg/mL (70–100% of donors are expected to react to this protein) and (ii) keyhole limpet hemocyanin, a recognized highly immunogenic protein.

Immunogenicity of lead clones was assessed in a T-cell proliferation assay using ProImmune Ltd REVEAL^®^ Immunogenicity System DC–T cell assay. The immunogenicity of each protein was determined by measuring the extent of T cells proliferation and by determining the number of immune-responsive individual donors treated with test proteins. Stimulation above background was determined using flow cytometric evaluation to count unlabeled, therefore proliferating T cells in each of the eight replicates for each sample. These values were then used to calculate percentage stimulation above background. Thus, the strength and frequency of response to each test sample could be used to calculate a response index (RI) value for each test protein (E06, BB10, BA11, BB11, and 2G) and control antigens (Figures 6B,C). Both the wild-type E06 and humanized variants had a very low RI compared to positive controls. Of the four humanized leads, clone 2G had the highest response. Clone BB11 gave a response, which was slightly higher than wild-type E06 with clones BA11 and BB10 having a slightly lower RI. These RI values were considered suitable for further development and similar to those of other therapeutic antibodies currently in the clinic and assayed using an identical protocol. Campath, Avastin, Humira, and Remicade had RI values of 0.26, 0.21, 0.14, and 0.49, respectively, and control RI values of 4 for KLH and 27.5 for PPD.

**Figure 7:**
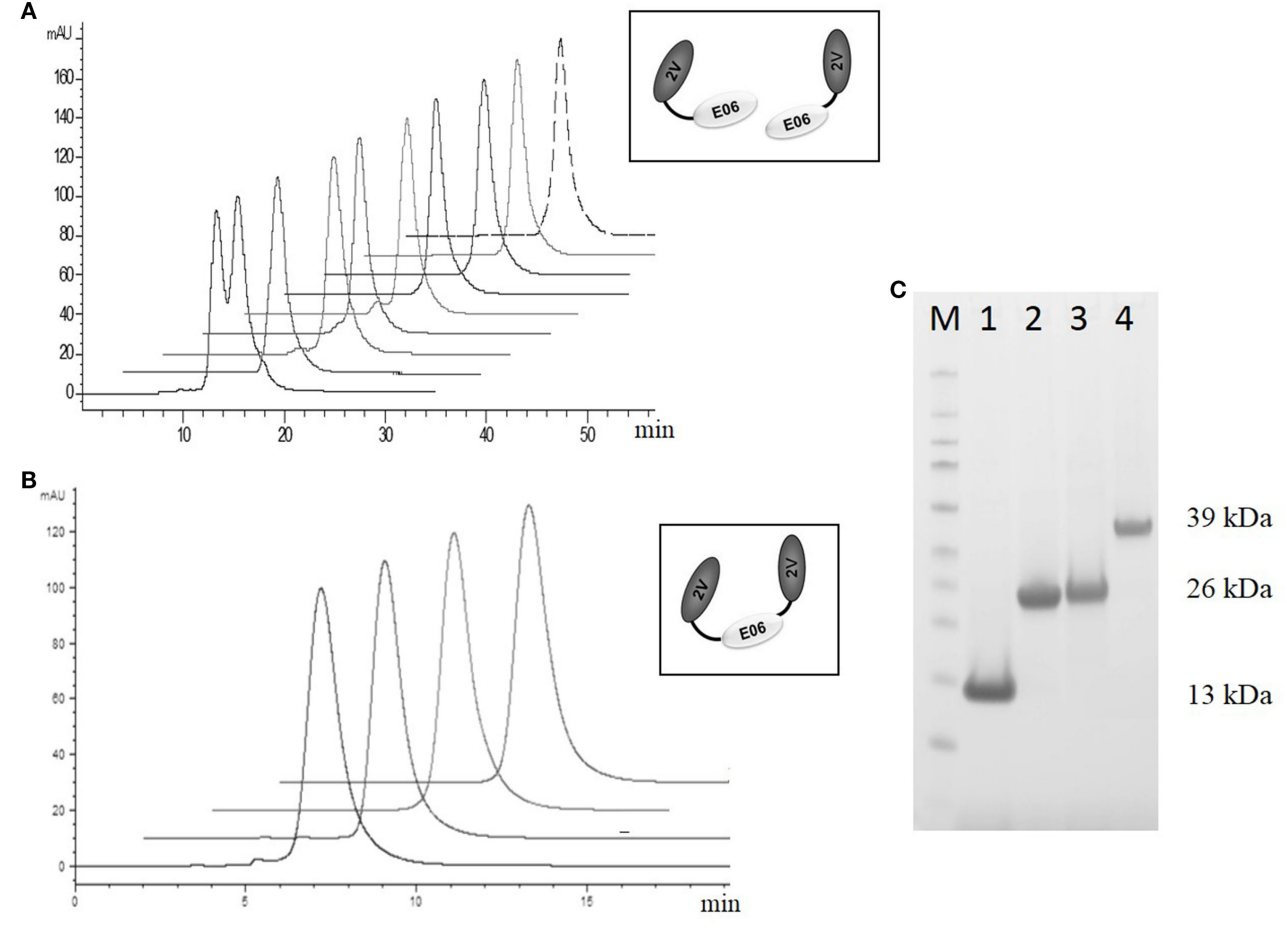
Analytical SEC and SDS PAGE analysis of purified lead humanized proteins reformatted as dimers and trimers. Purification and characterization of fusion proteins **(A)** Analytical SEC chromatograms of dimeric constructs, v1.10-2V, BB11-2V, 2V-BB11, BB10-2V, 2V-BB10, BA11-2V, 2V-BA11, E06-2V, and 2V-E06 overlaid and offset to aid comparisons. Note: The v1.10-2V protein elutes with two distinct peaks demonstrating the dimerization propensity seen originally with the monomeric v1.10 parental clone (Figure 2C). **(B)** Analytical SEC analysis of the trimeric constructs 2V-BB11-2V, 2V-E06-2V, 2V-BA11-2V, and 2V-BB10-2V. Chromatograms show trimeric proteins running as a single peak. **(C)** SDS PAGE analysis and Coomassie blue staining of approximately 5 µg of purified lead humanized protein BA11 as monomer and multimers. Lane M, Thermo Scientific Spectra Broad Range Protein Ladder; Lane 1, monomeric BA11; Lane 2, 2V-BA11; Lane 3, BA11-2V; Lane 4, 2V-BA11-2V.

Based on this antigenicity assessment, clone 2G was not chosen for further study while clones BB10, BA11 BB11, and wild-type E06 were reformatted as dimer or trimer genetic fusions and expressed with a second control VNAR called 2V. Originally isolated from the dogfish *Squalus acanthias*, 2V is part of a sequence database from this species and has no known target, making it an ideal control for these and other studies (23). When reformatted and expressed as amino or carboxyl terminal end dimeric fusion proteins (e.g., 2V-hE06), samples ran as single non-aggregated peaks when analyzed by analytical SEC (Figure 7A) with the exception of samples BB11-2V and 2V-BB10 that showed a minor peak eluting before the larger main peak, which may indicate the presence of some dimer in these samples. When the parental clone v1.10 was reformatted as a dimer with 2V, the resultant v1.10-2V protein retained its propensity to run as two peaks (Figure 7A). Formatting to produce the trimeric versions, 2V-E06-2V, 2V-BA11-2V, and 2V-BB10-2V also resulted in proteins which after purification ran predominantly as single peaks (Figure 7B) with BB11 containing trimeric construct, 2V-BB11-2V, showing a minor peak which eluted from the SEC column before the main peak. SDS PAGE analysis of monomer BA11, dimers 2V-BA11 and BA11-2V, and trimer 2V-BA11-2V confirmed that all migrated as single bands with significantly improved biophysical properties (Figure 7C).

### Affinity Measurements

**Figure 8:**
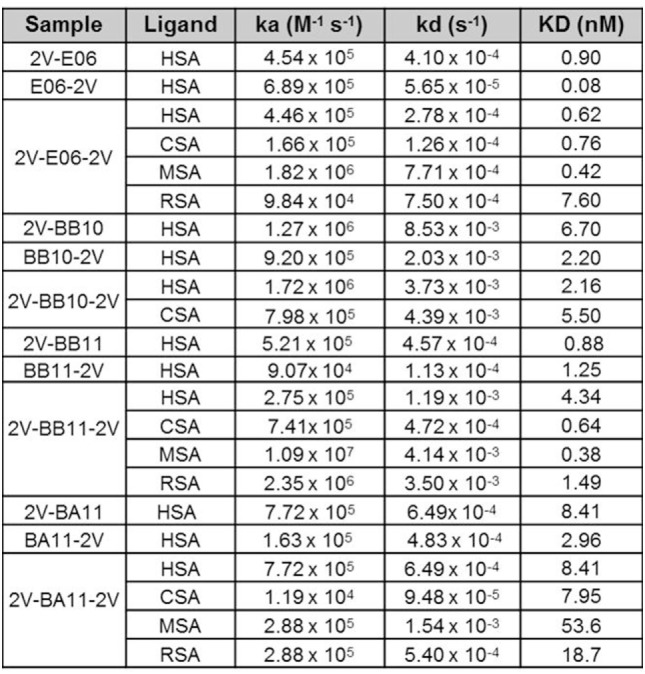
Affinity measurement of monomeric and reformatted dimeric and trimer humanized VNAR lead clones. BIAcore analyses of E06-2V, 2V-E06, 2V-E06-2V, BB10-2V, 2V-BB10, 2V-BB10-2V, BB11-2V, 2V-BB11, 2V-BB11-2V, 2V-BA11, BA11-2V, and 2V-BA11-2V against serum albumin from different species, human serum albumin, cynomolgus serum albumin, mouse serum albumin, and rat serum albumin, using a BIAcore instrument as described in the methods section. Kinetic measurements are summarized as averages from multiple runs.

Affinity measurements of purified anti-HSA VNAR and humanized VNAR monomers E06, 2G, BA11, BB11, and BB10 were determined by surface plasmon resonance using a T200 BIAcore instrument. The affinities for HSA, presented as a kinetic distribution plot (6A), were in the range of 0.58–24.9 nM. Affinities for rat and MSAs were in the range of 2.74–130 and 2.01–99 nM, respectively (Figure 6A). For all species tested, wild-type E06 had the highest affinity. The loss of binding of clone BB10 to rodent albumin could be attributed to the mutation in CDR3 and was therefore not taken forward as a candidate for further study. The eight dimeric constructs (BB11-2V, 2V-BB11, BB10-2V, 2V-BB10, BA11-2V, 2V-BA11, E06-2V, and 2V-E06) and four trimeric constructs (2V-BB11-2V, 2V-E06-2V, 2V-BA11-2V, and 2V-BB10-2V) bound HSA with high affinity including low picomolar values, 0.08–8.41 nM. In addition, binding and affinities for rat, mouse, and cynomolgus macaque albumins confirmed the utility of BB11 and BA11 as possible candidates for further clinical development (Figure 8).

### Rat PK Profile and Half-Life Determination of Candidate Clones

**Figure 9:**
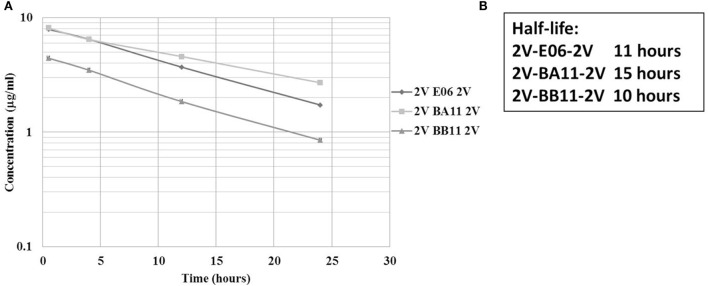
Rat pharmacokinetic (PK) profile and half-life determination of lead trimeric proteins. PK of trimeric VNAR constructs, 2V-BA11-2V, 2V-BB11-2V, and 2V-E06-2V domains delivered into Sprague Dawley rats *via* intravenous administration. **(A)** Graphical representation – mean data from four animals per group. **(B)** Calculated half-lives of administered trimeric proteins. Trimeric protein 2V-BB10-2V was not used in this study due to its loss of binding to rat serum albumin.

A previous PK study, in three animal species, demonstrated the ability of an albumin binding wild-type VNAR domain, E06, to extend the half-life of other proteins when administered as a molecular fusion (23). The present study looked at the ability of the humanized versions of E06, BB11, and BA11 to extend the circulating half-life of fusion proteins. Trimeric fusion proteins, 2V-BB11-2V, 2V-E06-2V, and 2V-BA11-2V, were expressed, purified, and characterized (Figures 6 and 8) prior to administration to rats at 1 mg/kg body weight. For this study, the sensitive and quantitative LC–MS techniques developed previously (23) were used to detect the presence of the test trimer VNAR fusion proteins in plasma samples. Circulating half-lives were determined of 11, 15, and 10 h for 2V-E06-2V, 2V-BA11-2V, and 2V-BB11-2V, respectively (Figure 9). Peptides derived from the albumin-binding domains E06, BA11, and BB11 as well as the 2V VNAR domain were detected, indicating that the domains remained stably linked for the duration of the study (results not shown).

### Stability

**Figure 10:**
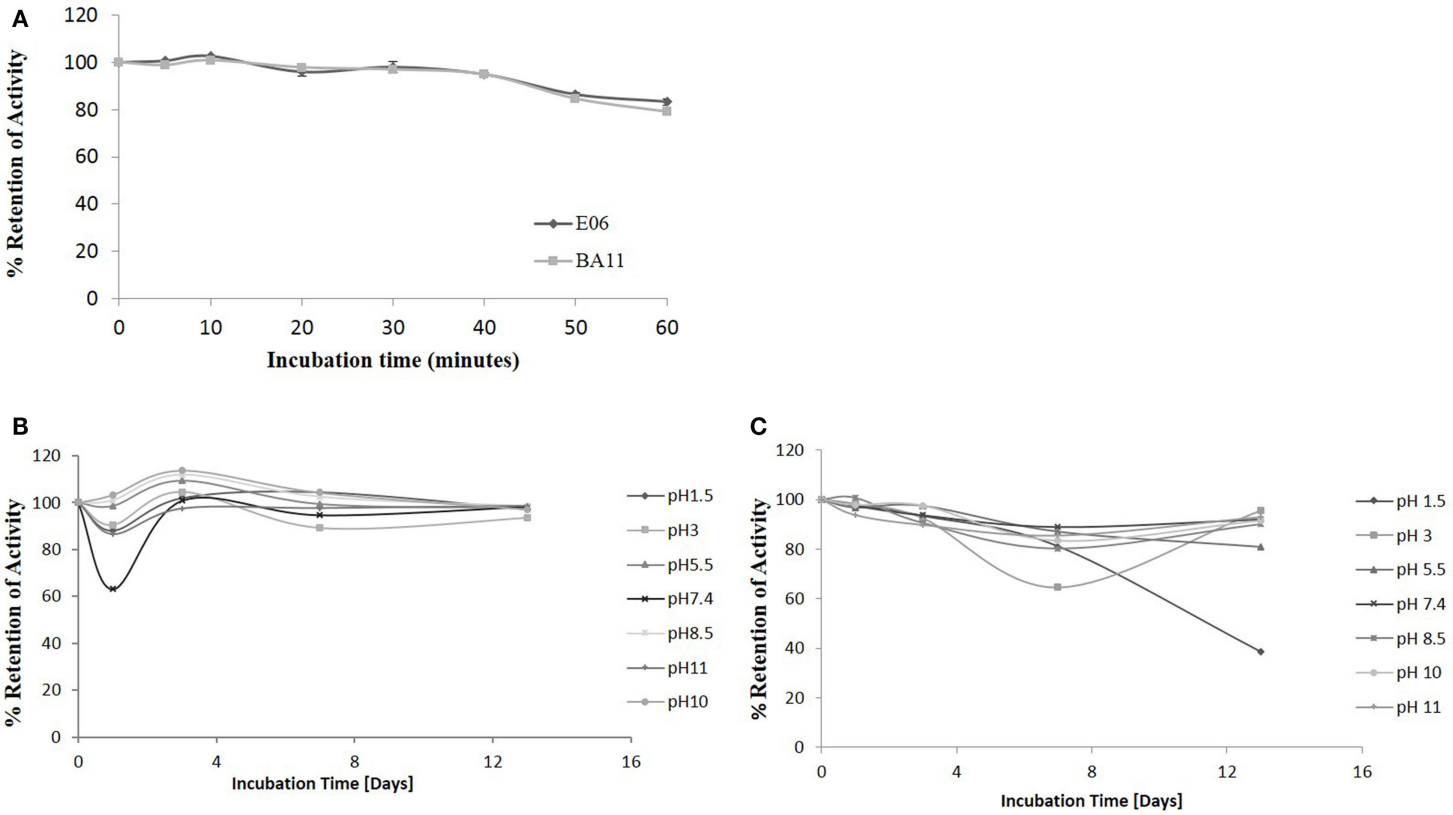
pH and thermo-stability. Stability analysis of the clones “parental” E06 and “candidate” BA11 to thermal and pH denaturation. **(A)** Residual antigen-binding activity was compared to a non-treated time zero control after test proteins were incubated at 100°C for 60 min. The data shown are the average of multiple replicates and a typical data set from 2 repeated experiments. **(B,C)** Residual antigen-binding activity following exposure of test proteins to a range of pH conditions for 14 days shown for E06 **(B)** and BA11 **(C)**.

The robust nature of VNAR monomeric proteins has been well documented (16, 32, 33). This robustness is characterized by the ability of VNARs to refold and bind cognate antigens after heating to high temperatures, up to 100°C, for extended periods of time. In this study, the ability of the shark VNAR parental protein E06 and candidate humanized variant BA11 were subjected to a challenge at both elevated temperatures and extremes of pH. Incubation at 100°C for 60 min (Figure 10A) resulted in both E06 and BA11 proteins retaining a high percentage (~80%) of antigen specific binding activity. When challenged by incubation at extremes of pH for up to 2 weeks, both proteins showed robust stability and retained the capacity to bind HSA after neutralization to pH 7.4 (Figures 10B,C). Only prolonged treatment of BA11 at pH 1.5 showed any drop off in antigen binding. Humanization of the E06 VNAR domain appears not to have had any marked detrimental or deleterious effect on the ability of the protein to refold correctly after denaturing challenge.

## Discussion

The utility of proteins as therapeutic agents is often compromised by their biophysical or biochemical properties which can lead to downstream processing issues including a propensity to precipitate or aggregate during purification and/or subsequent storage. Even if bio-processing is not an issue then a predisposition to elicit an adverse or immune response *in vivo* may also prevent further rapid development (34–36). While *in silico* modeling of VNAR domains predicts a low level of immunogenicity with similar values for those seen for a human Fc region (results not shown), we still embarked on a humanization strategy to accommodate any possible concerns from regulators. Immunogenicity testing of the parental E06 domain (Figure 6B) did in fact confirm these original *in silico* predictions. Earlier published efforts to humanize the HSA-binding, VNAR domain, E06 (24) produced a lead clone hE06v1.10, which unfortunately spontaneously dimerized (see Figure 1). A further version of humanized E06, based on human germline Kappa sequence DPK24, was also assessed, and although it was monomeric in solution and expressed well in recombinant systems, it unfortunately showed a greatly reduced affinity for target antigen. In an effort to improve these characteristics, both these humanized versions were subjected to a program of random mutagenesis (using error-prone PCR) and functional screening.

A panel of 15 clones (Figures 3B,C), demonstrating slow-off rates and strong ELISA binding, were chosen for further study and sequenced. Monomeric proteins for these clones were produced by transient expression in HEK 293 cells and EC_50_ values determined (Figures 4C,D). Clones BA11, BB11, BB10, and 2G emerged as the preferred “leads.” Only the EC_50_ values of clones generated by mutation of hE06v1.10 were close to the values of the starting HuE06v1.10 and E06 (picomolar range). The 2G clone however saw the greatest improvement in binding to 1.2 nM from a parental hE06v2.4 EC_50_ value of 171 nM (also confirmed by BIAcore). This 2G clone also had the greatest number of amino acid changes (4), although it is unclear from this work whether the improvement is the result of a single change or the four changes in concert.

Binding kinetics of the monomeric anti-HSA clones E06, BA11, BB11, BB10, and 2G was measured by surface plasmon resonance. The affinity of E06 was determined as 0.58 nM and that of huE06v1.10 at 6.4 nM (24). The affinities of the candidate humanized and mutated clones all fell within this range. For accelerated clinical development of the humanized clones, it is also important that they have retained the animal species cross-reactivity of E06 with comparable affinities. BA11, BB11, and 2G bound rat and MSAs with affinities between 2 and 130 nM. Interestingly, monomeric BB10 was no longer able to bind rodent albumin. This is due to the substitution of an isoleucine residue at position 90 in CDR3 with a larger hydrophobic phenylalanine residue. This hypothesis is supported by the published crystal structure (24) as isoleucine 90 contributes to hydrophobic intermolecular bonding between huE06 v1.10 and HSA. This lack of species cross-reactivity precluded BB10’s use as a candidate clone for future clinical development.

Analysis of a larger number of mutated sequences from both libraries, performed as a part of their quality control, revealed that multiple changes of up to four amino acid positions were observed (data not shown). The four lead clones had a change in CDR3, indeed in clones BB10 and BB11, this was the only region where a change of residue occurs. For BB11, the glycine at position 87 was replaced by an alanine residue. BA11 also has a change at position 87 to a serine and also at position 78 where a phenylalanine is replaced by a serine. Clone 2G, which comes from the v2.4 background, has four mutations from the parental v2.4: asparagine 45 to a lysine, serine 52 to a glycine, leucine 73 to a methionine and a change in CDR3 where glycine 87 is mutated to a serine. The result of these mutations was a recovery of binding affinity but may have also contributed to the increased immunogenicity seen for this clone (Figure 6). Interestingly, clones 1H and 5F, from the v2.4 background, both had a substitution at position 88 from a valine to an alanine. Clone 5F also had a mutation at position 63 in HV4 from a threonine to an asparagine. Previous work has noted that although containing no contact residues, the HV4 region of E06 contributed to the binding of albumin by packing against contact residues of CDR1 (24). This second mutation in the HV4 region of clone 5F may explain the improvement noted in EC_50_ values, which decreased from 82 nM (1H) to 18 nM (5F) and supports the theory that HV4 packs against the CDR1 region, thereby positioning amino acid residues of the CDR1 loop in contact with albumin. The highly selected change of CDR3 residue 87 (BB11, BA11, and 2G) is not an antigen specific contact residue (24), and therefore, retention of binding specificity and affinity is unsurprising. However, it does appear that a change at this position (or close by at position 90 for BB10) has resolved the dimerization bio-processing issue identified as a problem in the parental backbone v1.10 (Figure 2). While this phenomenon has been seen previously in antibody CDR regions, where particular motifs contribute to the multimerization of antibody proteins in solution (37, 38), we were surprised that such a small change from a glycine to a serine or even alanine could result in the dramatic improvement in manufacturability of the expressed protein.

Human immune response to bio-therapeutic proteins is notoriously difficult to predict and varies from individual to individual. It has been reported that proteins having high homology with human proteins or humanized proteins have reduced immunogenicity (35), but this cannot simply be presumed to be the case. No assay is capable of definitively predicting *in vivo* antigenicity with the only real test being observations from “in human” clinical studies (35). However, *in vitro* testing combined with bench-marking against existing clinical assets can provide a reasonable level of comfort when selecting candidate molecules for costly late stage pre-clinical studies. Humanized E06 VNAR domains BA11, BB11, BB10, and 2G showed very low RI values in a human donor T-cell proliferation assay using ProImmune Ltd. REVEAL^®^ Immunogenicity System DC–T cell assay. Importantly, the RI values were comparable to those obtained when Campath, Avastin, Humira, and Remicade were investigated using the same assay. In fact, when control values are also compared, the humanized VNAR, BB10, had a relative RI slightly below those determined for this panel of well-known clinical agents and chosen lead BA11 has a lower RI than Remicade and is similar to humanized antibodies Campath and Avastin.

Based on a combination of affinity, antigenicity, species cross-reactivity, and expressability, BA11 and BB11 (together with E06 and BB10 controls) were reformatted as dimeric and trimeric fusion proteins using a naive control VNAR 2V. By SDS PAGE, these proteins migrated as single bands with predicted molecular masses (Figure 7C) and confirmed that the bio-processing and affinity improvements seen for the monomeric humanized mutants was retained when they were assembled in a more relevant therapeutic format (fusions at the carboxyl terminal, amino terminal fusion, or both). Indeed 2V-BA11 protein was concentrated in PBS to therapeutic levels of over 50 mg/mL without precipitation, a favorable property for drug formulation, and retained a single-peak, SEC profile (data not shown).

Albumin half-life in the systemic circulation is prolonged by the FcRn recycling process (39, 40). In an early publication, monomeric and multimeric VNAR constructs containing the wild-type E06 albumin-binding domain showed a very impressive extended half-life equivalent to that of the species-specific (rodent or non-human primates) serum albumin (23). In this study, the functional ability of the humanized albumin-binding domains BA11 and BB11 and the wild-type E06 to extend circulating half-life, formatted as fusion proteins with the 2V VNAR domain (as trimers), was examined in a suitable rodent model. The humanized variants had a half-life (2V-BA11-2V 15 h and 2V-BB11-2V 10 h) equivalent to the parental control (2V-E06-2V 11 h), thereby confirming that the humanized domains remained bound to RSA during the FcRn recycling process.

Based on the accumulated data from affinity, analytical SEC profile, expressability, protein bio-processing quality, immunogenicity, and rodent-half-life studies, BA11 emerged as the clear candidate molecule for further study. To avoid confusion, it is worth noting that recent commercial literature often refers to humanized VNAR as soloMERs™ (22) and BA11 as NDure™.

Single-domain antibodies from shark (16, 32, 33) and camelid (41–43) species have an ability to refold and bind antigen after thermal and chemical denaturation. Here, the candidate clone BA11 was subjected to extreme thermal and pH challenge. Both the monomeric wild-type E06 and candidate clone showed a remarkable ability to bind cognate antigen after temperature or pH induced unfolding, confirming that functional binding and surprisingly functional stability (which had been ignored as part of clone “culling”) had been retained through the mutation and selection process.

Naturally occurring protein binding domains with the potential for development as bio-therapeutic drugs are being investigated many research and development laboratories of the pharmaceutical industry. In this article, we have detailed the successful humanization of an albumin binding domain isolated from shark VNAR library. This domain, E06, was humanized in a manner that facilitated the retention of high affinity and specificity for cognate antigen with low immunogenicity and the hallmark high stability of native VNARs. *In vivo* PK studies have proven this final clinical domain BA11 (NDure™) to have broad utility as an enabling product for increased systemic exposure across multiple therapeutic modalities while retaining the benefit of small size and can be formulated at concentrations concurrent with clinically approved mAbs.

## Ethics Statement

All animal studies were conducted by Charles River (Elpinstone Research Centre, Elphinstone, Tranent EH33 2NE) in accordance with their regulated procedures.

## Author Contributions

CB is the PI on the project and managed and led the team. AP is the co-PI. JS, MM, MC, OU, MK, and GD are the senior scientists who conducted the work. TB, DC, and KS developed and ran the LC/MS.

## Conflict of Interest Statement

The authors declare that the research was conducted in the absence of any commercial or financial relationships that could be construed as a potential conflict of interest.

## Abbreviations

IgNAR, immunoglobulin new antigen receptor; VNAR, variable domain of shark new antigen receptor; CDR, complementarity-determining region; FW, framework HV, hypervariable region; PK, pharmacokinetics; HSA, human serum albumin.

## References

[1] Padlan EA. A possible procedure for reducing the immunogenicity of antibody variable domains while preserving their ligand-binding properties. Mol Immunol (1991) 28:489–98. 10.1016/0161-5890(91)90163-E 1905784

[2] Pelat T Bedouelle H Rees AR Crennell SJ Lefranc M Thullier P. Germline humanization of a non-human primate antibody that neutralizes the anthrax toxin, by in vitro and in silico engineering. J Mol Biol (2008) 384:1400–7. 10.1016/j.jmb.2008.10.033 18976662

[3] Robert R Streltsov VA Newman J Pearce LA Wark KL Dolezal O. Germline humanization of a murine Abeta antibody and crystal structure of the humanized recombinant Fab fragment. Protein Sci (2010) 19:299–308. 10.1002/pro.312 20014445 PMC2865728

[4] Cheung NV Guo H Hu J Tassev DV Cheung IY. Humanizing murine IgG3 anti-GD2 antibody m3F8 substantially improves antibody-dependent cell-mediated cytotoxicity while retaining targeting in vivo. Oncoimmunology (2012) 1:477–86. 10.4161/onci.19864 22754766 PMC3382886

[5] Zhao H Verma D Li W Choi Y Ndong C Fiering SN Depletion of T cell epitopes in lysostaphin mitigates anti-drug antibody response and enhances antibacterial efficacy in vivo. Chem Biol (2015) 22:629–39. 10.1016/j.chembiol.2015.04.017 26000749 PMC4441767

[6] Reichert JM Antibodies to watch in 2017. MAbs (2016) 9(2):167–81. 10.1080/19420862.2016.1269580 27960628 PMC5297518

[7] Demidem A Lam T Alas S Hariharan K Hanna N Bonavida B. Chimeric anti-CD20 (IDEC-C2B8) monoclonal antibody sensitizes a B cell lymphoma cell line to cell killing by cytotoxic drugs. Cancer Biother Radiopharm (1997) 12:177–86. 10.1089/cbr.1997.12.177 10851464

[8] Goldenberg MM. Trastuzumab, a recombinant DNA-derived humanized monoclonal antibody, a novel agent for the treatment of metastatic breast cancer. Clin Ther (1999) 21:309–18. 10.1016/S0149-2918(00)88288-0 10211534

[9] Salgaller ML Technology evaluation: bevacizumab, Genentech/Roche. Curr Opin Mol Ther (2003) 5:657–67. 14755893

[10] Kovaleva M Ferguson L Steven J Porter A Barelle C Shark variable new antigen receptor biologics – a novel technology platform for therapeutic drug development. Expert Opin Biol Ther (2014) 14:1527–39. 10.1517/14712598.2014.937701 25090369

[11] Gebauer M Skerra A. Engineered protein scaffolds as next-generation antibody therapeutics. Curr Opin Chem Biol (2009) 13:245–55. 10.1016/j.cbpa.2009.04.627 19501012

[12] Scolnik PA. mAbs: a business perspective. MAbs (2009) 1:179–84. 10.4161/mabs.1.2.7736 20061824 PMC2725420

[13] Sexton K Tichauer K Samkoe KS Gunn J Hoopes PJ Pogue BW. Fluorescent affibody peptide penetration in glioma margin is superior to full antibody. PLoS One (2013) 8:e60390. 10.1371/journal.pone.0060390 23593208 PMC3625207

[14] Xiang D Zheng C Zhou SF Qiao S Tran PH Pu C Superior performance of Aptamer in tumor penetration over antibody: implication of Aptamer-based theranostics in solid tumors. Theranostics (2015) 5:1083–97. 10.7150/thno.11711 26199647 PMC4508498

[15] Greenberg AS Hughes AL Guo J Avila D McKinney EC Flajnik MF A novel “chimeric” antibody class in cartilaginous fish: IgM may not be the primordial immunoglobulin. Eur J Immunol (1996) 26:1123–9. 10.1002/eji.1830260525 8647177

[16] Dooley H Flajnik MF Porter AJ. Selection and characterization of naturally occurring single-domain (IgNAR) antibody fragments from immunized sharks by phage display. Mol Immunol (2003) 40:25–33. 10.1016/S0161-5890(03)00084-1 12909128

[17] Goodchild SA Dooley H Schoepp RJ Flajnik M Lonsdale SG. Isolation and characterisation of Ebolavirus-specific recombinant antibody fragments from murine and shark immune libraries. Mol Immunol (2011) 48:2027–37. 10.1016/j.molimm.2011.06.437 21752470

[18] Müller MR O’Dwyer R Kovaleva M Rudkin F Dooley H Barelle CJ. Generation and isolation of target-specific single-domain antibodies from shark immune repertoires. Methods Mol Biol (2012) 907:177–94. 10.1007/978-1-61779-974-7_9 22907351

[19] Streltsov VA Varghese JN Carmichael JA Irving RA Hudson PJ Nuttall SD. Structural evidence for evolution of shark Ig new antigen receptor variable domain antibodies from a cell-surface receptor. Proc Natl Acad Sci U S A (2004) 101:12444–9. 10.1073/pnas.0403509101 15304650 PMC515081

[20] Dooley H Flajnik MF. Shark immunity bites back: affinity maturation and memory response in the nurse shark, *Ginglymostoma cirratum*. Eur J Immunol (2005) 35:936–45. 10.1002/eji.200425760 15688348

[21] Dooley H Flajnik M Antibody repertoire development in cartilaginous fish. Dev Comp Immunol (2006) 30:43–56. 10.1016/j.dci.2005.06.022 16146649

[22] Barelle C Muller MR Calabro V Bikker J Steven J Tchistiakova L Single Domain Binding Molecule. U.S.Patent No US20170096475 A1. Washington, DC: U.S. Patent and Trademark Office (2016).

[23] Müller MR Saunders K Grace C Jin M Piche-Nicholas N Steven J Improving the pharmacokinetic properties of biologics by fusion to an anti-HSA shark VNAR domain. MAbs (2012) 4:673–85. 10.4161/mabs.22242 23676205 PMC3502234

[24] Kovalenko OV Olland A Piche-Nicholas N Godbole A King D Svenson K Atypical antigen recognition mode of a shark immunoglobulin new antigen receptor (IgNAR) variable domain characterized by humanization and structural analysis. J Biol Chem (2013) 288:17408–19. 10.1074/jbc.M112.435289 23632026 PMC3682541

[25] Finlay WJ Cunningham O Lambert MA Darmanin-Sheehan A Liu X Fennell BJ Affinity maturation of a humanized rat antibody for anti-RAGE therapy: comprehensive mutagenesis reveals a high level of mutational plasticity both inside and outside the complementarity-determining regions. J Mol Biol (2009) 388:541–58. 10.1016/j.jmb.2009.03.019 19285987

[26] Vaughan TJ Williams AJ Pritchard K Osbourn JK Pope AR Earnshaw JC Human antibodies with sub-nanomolar affinities isolated from a large non-immunized phage display library. Nat Biotechnol (1996) 14:309–14. 10.1038/nbt0396-309 9630891

[27] Fennell BJ Darmanin-Sheehan A Hufton SE Calabro V Wu L Muller MR Dissection of the IgNAR V domain: molecular scanning and orthologue database mining define novel IgNAR hallmarks and affinity maturation mechanisms. J Mol Biol (2010) 400:155–70. 10.1016/j.jmb.2010.04.061 20450918

[28] Leonard P Säfsten P Hearty S McDonnell B Finlay W O’Kennedy R. High throughput ranking of recombinant avian scFv antibody fragments from crude lysates using the Biacore A100. J Immunol Methods (2007) 323:172–9. 10.1016/j.jim.2007.04.010 17532001

[29] Cummins E Luxenberg DP McAleese F Widom A Fennell BJ Darmanin-Sheehan A A simple high-throughput purification method for hit identification in protein screening. J Immunol Methods (2008) 339:38–46. 10.1016/j.jim.2008.07.016 18760282

[30] Huh S Do H Lim H Kim D Choi S Song H Optimization of 25kDa linear polyethylenimine for efficient gene delivery. Biologicals (2007) 35:165–71. 10.1016/j.biologicals.2006.08.004 17084092

[31] Backliwal G Hildinger M Hasija V Wurm FM. High-density transfection with HEK-293 cells allows doubling of transient titers and removes need for a priori DNA complex formation with PEI. Biotechnol Bioeng (2008) 99:721–7. 10.1002/bit.21596 17680657

[32] Shao C Secombes CJ Porter AJ. Rapid isolation of IgNAR variable single-domain antibody fragments from a shark synthetic library. Mol Immunol (2007) 44:656–65. 10.1016/j.molimm.2006.01.010 16500706

[33] Liu JL Zabetakis D Brown JC Anderson GP Goldman ER. Thermal stability and refolding capability of shark derived single domain antibodies. Mol Immunol (2014) 59:194–9. 10.1016/j.molimm.2014.02.014 24667069

[34] Koren E Smith HW Shores E Shankar G Finco-Kent D Rup B Recommendations on risk-based strategies for detection and characterization of antibodies against biotechnology products. J Immunol Methods (2008) 333:1–9. 10.1016/j.jim.2008.01.001 18275969

[35] Attarwala H. TGN1412: from discovery to disaster. J Young Pharm (2010) 2:332–6. 10.4103/0975-1483.66810 21042496 PMC2964774

[36] Chirmule N Jawa V Meibohm B. Immunogenicity to therapeutic proteins: impact on PK/PD and efficacy. AAPS J (2012) 14:296–302. 10.1208/s12248-012-9340-y 22407289 PMC3326159

[37] Dudgeon K Famm K Christ D. Sequence determinants of protein aggregation in human VH domains. Protein Eng Des Sel (2009) 22:217–20. 10.1093/protein/gzn059 18957405

[38] Wang X Das TK Singh SK Kumar S. Potential aggregation prone regions in biotherapeutics: a survey of commercial monoclonal antibodies. MAbs (2009) 1:254–67. 10.4161/mabs.1.3.8035 20065649 PMC2726584

[39] Chaudhury C Brooks CL Carter DC Robinson JM Anderson CL Albumin binding to FcRn: distinct from the FcRn-IgG interaction. Biochemistry (2006) 45:4983–90. 10.1021/bi052628y 16605266

[40] Kim J Bronson CL Hayton WL Radmacher MD Roopenian DC Robinson JM Albumin turnover: FcRn-mediated recycling saves as much albumin from degradation as the liver produces. Am J Physiol Gastrointest Liver Physiol (2006) 290:G352–60. 10.1152/ajpgi.00286.2005 16210471

[41] Van der Linden R Frenken L De Geus B Harmsen M Ruuls R Stok W Comparison of physical chemical properties of llama V HH antibody fragments and mouse monoclonal antibodies. Biochim Biophys Acta (1999) 1431:37–46. 10.1016/S0167-4838(99)00030-8 10209277

[42] Dumoulin M Conrath K Van Meirhaeghe A Meersman F Heremans K Frenken LG Single-domain antibody fragments with high conformational stability. Protein Sci (2002) 11:500–15. 10.1110/ps.34602 11847273 PMC2373476

[43] Dolk E van der Vaart M Lutje Hulsik D Vriend G de Haard H Spinelli S Isolation of llama antibody fragments for prevention of dandruff by phage display in shampoo. Appl Environ Microbiol (2005) 71:442–50. 10.1128/AEM.71.1.442-450.2005 15640220 PMC544197

